# The Influence of Zinc Stearate Complexes on the Sulfur Vulcanization of Ethylene–Propylene–Diene Monomer

**DOI:** 10.3390/polym17212875

**Published:** 2025-10-28

**Authors:** Krzysztof Kaczewiak, Piotr Głąb, Magdalena Maciejewska

**Affiliations:** 1 Interdisciplinary Doctoral School, Lodz University of Technology, 116 Żeromskiego Street, 90543 Lodz, Poland; 2 Hutchinson Poland Sp z o.o., 130 Kurczaki Street, 90543 Lodz, Poland; 3 Institute of Polymer and Dye Technology, Lodz University of Technology, 16 Stefanowskiego Street, 90543 Lodz, Poland

**Keywords:** EPDM, cross-linking density, compression set, elastomers, carbon footprint

## Abstract

This article explores the influence of synthesized zinc stearate (ZnSt) complexes with different molar ratios of zinc oxide (ZnO) and stearic acid (StA) on the sulfur vulcanization and properties of the ethylene–propylene–diene monomer (EPDM). The aim of study was to reduce the amount of ZnO in elastomer composites without affecting their compression set. The research involves synthesizing zinc complexes, including the addition of sulfur, and using them as activators in EPDM compounds. The synthesized products were characterized using DSC and FTIR to identify their composition and thermal properties. The results of the compression set and equilibrium swelling tests indicated that ZnO was used in excess as an activator. Zinc complexes allowed for a significant reduction in zinc content compared to ZnO. The stoichiometric salt of ZnO and StA was the most effective in terms of vulcanization parameters and the compression set. Non-stoichiometric complexes were less effective, proportionally to their content. SEM images showed that the dispersion of components was relatively homogeneous in vulcanizates with active ZnO and commercial ZnSt. However, the blooming of ZnSt from vulcanizates was observed. Thus, using synthesized materials could reduce the zinc content in EPDM composites while maintaining good properties, but a further reduction is needed to avoid blooming.

## 1. Introduction

Cross-linked rubber is a material with exceptional elastic properties, which plays a key role in many areas of our lives. Its ability to stretch and return to its original shape makes it irreplaceable in the production of tires, seals, medical gloves, and many other rubber products. Thanks to its unique properties, rubber is a material that has revolutionized many industries and continues to play a key role in the modern world.

The production of rubber composites is a complex process that requires precision and advanced technology to obtain products of high quality and durability. Rubber composites consist of both rubber and a number of other functional additives, e.g., fillers, plasticizers, activators, accelerators, or antioxidants, which improve their processing and facilitate properties of the vulcanizates. One of the substances widely used in the vulcanization process is zinc oxide (ZnO) [1,2]. It acts as an activator, speeding up the vulcanization reaction by activating the vulcanization accelerators [3]. When combined with stearic acid (StA), it increases the effectiveness of sulfur in the vulcanization process. Moreover, adding ZnO to a rubber compound improves the tensile strength, abrasion resistance, and elasticity of vulcanizates. Due to the key role of ZnO in elastomer technology, it is estimated that over 50% of ZnO production is consumed by the rubber industry [4,5]. Other industries consuming ZnO include agriculture, ceramics, and concrete. ZnO is also used on a smaller scale in the electronics industry and cosmetics production. ZnO used in the rubber industry is most often obtained in a metallurgical process by roasting zinc ore in the presence of air [6,7]. Alternatively, ZnO can be obtained by using a number of chemical methods, not always applicable to the industrial-scale production of ZnO due to the low efficiency of synthesis and economics [8]. ZnO production also generates carbon dioxide (CO_2_) and other greenhouse gas emissions, mainly due to the energy processes involved in roasting ores or drying and roasting the products of chemical synthesis methods. Depending on the method used, the carbon footprint of ZnO production can vary [9,10]. Metallurgical processes are usually more energy-intensive and generate larger CO_2_ emissions. On the other hand, chemical methods can be less energy-intensive, but require the use of other reagents, which also has an impact on the environment. The production of ZnO, regardless of the method, is therefore a harmful process for the natural environment; hence, it is justified to look for possibilities of reducing its consumption in various industries, especially in the case of the rubber industry, which is the main consumer of this material.

Regarding the rubber industry, ZnO is considered as the best activator for sulfur cross-linking due to its higher reaction kinetics and promotion of short sulfur cross-links that increase the overall cross-linking density of vulcanized rubber [11,12]. It also acts as a filler and scavenger of harmful reaction products, a cure agent for halogenated or carboxylated rubbers, and even provides protection from molded product shrinkage or mold fouling. In addition, ZnO has a significant effect on some of the physical properties of vulcanizates. Owing to its poor dispersibility in the rubber matrix, ZnO demands a high dosage to ensure efficient vulcanization. From an ecological point of view, a high amount of ZnO raises a concern about its release due to the exploitation of rubber products and its toxicity for the environment [1]. Generally, ZnO is considered as a substance safe for humans, but based on the international standard, i.e., Globally Harmonized System of Classification and Labelling of Chemicals (GHS), it is classified as H400: very toxic to aquatic life. Hence, for the sake of the environment, it is recommended to reduce the amount of ZnO in rubber products. It should be noticed that, when ZnO is used in vulcanization in the form of microsized ZnO crystal particles, only a reduced portion is able to react with the curing compounds, leading to a residual amount of unreacted ZnO in the rubber matrix. This unreacted ZnO can penetrate into groundwater while rubber products are stored in landfills, or in the form of dust, which is generated as a result of rubbing tires on the road surface. In the case of tires, a consequence of the wear of tire treads is zinc leaching, which may generate an excess of zinc concentration in the environment, potentially increasing in the future due to the widespread and expanding use of ZnO. This makes the topic of ZnO reduction very important for the rubber industry, as one of the largest users of ZnO.

The efficiency of ZnO in vulcanization and its influence on the properties of rubber composites strongly depend on the dispersion degree of ZnO particles in the elastomer matrix. Obtaining a good dispersion of ZnO in the elastomer matrix, and consequently an availability of ZnO particles for reaction with other components of the cross-linking system, can significantly improve its activity in the cure process and the final mechanical properties of the vulcanizates. Sreethu and Naskar [11] compared the activation effectiveness and impact on mechanical properties and the cure characteristics of different types of ZnO, i.e., conventional, active, and nanopowder, in the vulcanization of natural rubber filled with carbon black. Their investigation showed that a more homogeneous dispersion and higher surface area of ZnO can help to reduce its content without decreasing the mechanical properties of the rubber composites. The addition of fatty acids, such as StA, also plays a significant role in vulcanization [1,13]. They improve the dispersion of ZnO particles in the rubber matrix by boosting the contact surface of zinc ions with vulcanization accelerators. ZnO reacts with StA, forming zinc stearate (ZnSt), which is assumed as an essential cure activator. Junkong et al. [14] performed in situ research to study the activity of different fatty acids in comparison with StA and ZnSt in the vulcanization of isoprene rubber. It was shown that ZnO creates 2:2 bidentate zinc/stearate complexes, with StA also having some hydroxyl groups. These complexes act as catalysts to accelerate the sulfur cross-linking reactions in the rubber matrix. Heideman et al. [15] studied various metal oxides and a clay loaded with Zn^2+^ ions as a solution that can help reduce the zinc content in rubber composites. Hence, zinc stearate, zinc 2-ethylhexanoate, and zinc m-glycerolate were employed instead of ZnO to activate the sulfur vulcanization of the ethylene–propylene–diene monomer (EPDM) and styrene–butadiene (SBR) rubber. Zinc m-glycerolate, with the highest amount of zinc per 1 g, showed the highest activity in the vulcanization. However, no reduction in the zinc amount was provided compared to rubber compounds cured with ZnO, since the authors of the publication used zinc complexes in an amount equivalent to the content of zinc ions in the amount of ZnO used. Therefore, the possibility of reducing the amount of zinc in rubber products was not investigated, but only the possibility of replacing ZnO with other zinc compounds. Przybyszewska et al. [16] reported that the use of organic zinc activators in the form of zinc salts or zinc complexes with 1,3-diketones, depending on their structure, allowed for a reduction in the amount of zinc in rubber compounds by 70–90 wt.% compared to those containing ZnO and stearic acid. However, vulcanizates with a lower cross-link density compared to those containing ZnO were obtained, which can have a significant impact on their mechanical properties, e.g., the compression set, which is crucial for rubber seals. Thus, the results reported in the literature are quite optimistic, but they need to be investigated further due to a lack of clear knowledge of all variables that affect the vulcanization process.

**Figure 1:**
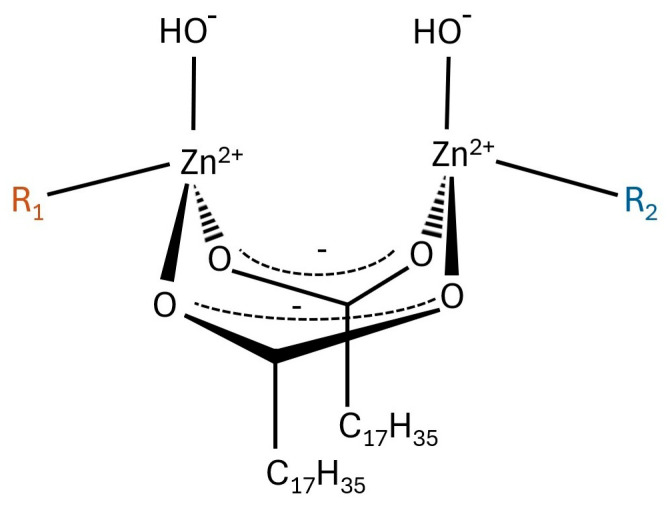
Binuclear type bridging bidentate zinc/stearate complex, where R_1_ and R_2_ are water and/or a rubber segment, based on [17].

Considering the vulcanization, there is a lot of information in the literature about ZnO and StA complex formation. Scotti [12] prepared an extensive review, which indicated that stoichiometric ZnSt is not an efficient activator of sulfur vulcanization. However, Junkong et al. [14] observed that non-stoichiometric salts of ZnO and fatty acids can effectively activate vulcanization. It was found that, at high temperatures, a dominant structure of the product formed in the ZnO and StA reaction was a bridging bidentate zinc/stearate complex with a molar ratio of 2:2. It involved two zinc ions bridged by two stearate (StA) ligands, where each stearate molecule coordinated to both zinc ions with its carboxylate group. This structure was represented as (Zn_2_(μ-O_2_CC_17_H_35_)_2_)^2+^(OH–)_2_·R_1_R_2_, where R_1_ and R_2_ can be water molecules or rubber segments, respectively. The most likely structure of the binuclear type bridging bidentate zinc/stearate complex is presented in Figure 1 [17].

A good example of a non-stoichiometric complex of ZnO and StA was also described by Setianto et al. [18]. The aim of this study was to produce ZnSt; however, ZnO and StA were mixed in non-stoichiometric proportions. Instead of 2 moles of StA per 1 mole of ZnO, the synthesis involved 3 moles of ZnO per 1 mole of StA, which resulted in a 6-fold excess of ZnO. Surprisingly, the tensile strength and hardness of the vulcanizates obtained by replacing ZnO and StA with a synthesis product showed significant improvement. This suggests that the non-stoichiometric complex of ZnO with StA can be potentially as active as the high amount of active ZnO, which is typically used in rubber compounds in the amount of 3 phr. Hence, the use of non-stoichiometric zinc complexes with StA can potentially contribute to the reduction in the amount of zinc in elastomer composites compared to those containing active ZnO, or even to the elimination of active ZnO. On the other hand, there is no information in the literature about sulfur-ZnO complex formation during the vulcanization of rubber compounds. The past research showed that there is a reaction between ZnO and sulfur, with the creation of zinc sulfide (ZnS), which is deposited on the surface of the vulcanization molds [19]. This proves that, during the vulcanization of rubber, like EPDM, some amount of ZnO is transformed into ZnS, which can influence the rubber cross-linking density and consequently the vulcanizate properties [3]. Thus, in this work, we synthesized zinc complexes using different molar ratios of ZnO:StA, while additionally introducing sulfur into some of these complexes. Standard zinc stearate (ZnSt) contains two moles of monovalent StA anions for one mole of divalent cation of zinc. Taking into consideration that, in the literature, there are plenty of studies confirming the formation of non-stoichiometric complexes between stearic acid and ZnO, we decided to check how they affect the curing characteristics of rubber composites and properties of the vulcanizates. During previously conducted research, we have also observed that heating sulfur with ZnO in a masterbatch before adding an accelerator pack improved the functional parameters of vulcanizates, i.e., the compression set, despite the cross-linking density reduction [20]. Therefore, in this research, samples with sulfur were also prepared. Moreover, syntheses in the presence of sulfur were performed to verify the potential formation of complexes between carboxylic acid, sulfur, and ZnO. Then, we used the obtained synthesis products as activators of sulfur vulcanization as an alternative to systems containing ZnO, StA, and sulfur separately. We believed that the use of these complexes would reduce the amount of ZnO in elastomer composites, and thus the harmfulness of ZnO to the environment and the carbon footprint associated with its application.

## 2. Materials and Methods

### 2.1. Materials

The masterbatch of the ethylene-propylene-diene monomer compound with the hardness of 74 Shore A (74ShA EPDM) used for the study was a serial product used on extrusion. This compound was vulcanized with a sulfur cure system. Its composition is not revealed due to the Hutchinson confidentiality policy. In case of cure system adjustments, the 74ShA compound was mixed in the form of a masterbatch that did not contain any components of the curing system, i.e., sulfur, ZnO, or StA. The accelerators were commercially available predispersions purchased from Konimpex Chemicals Sp. Z o.o. (Konin, Poland), with the following concentration of active ingredient: caprolactam disulfide (CLD)—80%, 2,2’-benzothiazyl disulfide (MBTS)—70%, N-cyclohexyl-2-benzothiazole sulfenamide (CBS)—80%, and zinc dialkyldithiophosphate (ZDTP)—50%. Zinc oxide with a specific surface area of 10 m^2^/g (Huta Będzin, Będzin, Poland) and active zinc oxide with a specific surface area of 45 m^2^/g (Silox India, Vadodara, India), together with stearic acid (Uniqema, Goole, UK), were used to synthesize zinc complexes. Zinc stearate with a purity > 98.5% was purchased from Warchem (Trakt Brzeski, Poland), while sulfur was manufactured by Siarkopol (Tarnobrzeg, Poland).

### 2.2. Synthesis of Zinc Complexes and Salts

The sets of synthesis products were prepared in a flask heated with methyl silicone oil to 160 °C. Mixing was ensured by a magnetic stirrer. The synthesis station is presented in Supplementary Materials (Figure S1). The mixtures were prepared according to the recipes expressed in moles in Table 1.

For samples 1 to 8 given in Table 1, ZnO was added to the melted technical StA and stirred at 160 °C. After a turbulent synthesis reaction with the release of water vapor, sulfur was added according to the recipes presented in Table 1, and all samples were further heated and stirred for 45 min. Samples 9 and 10 were prepared according to the same recipe as samples 7 and 8 but differed in the order of dosing the individual components. Sulfur was added to the melted stearic acid, and, after it had melted, active ZnO was added. After the synthesis reaction had taken place, the mixture was stirred and heated for 45 min at 160 °C.

Stearic acid used for the synthesis was a standard material used in rubber industry, containing 57% of stearic acid (C_17_H_35_COOH), 35% of palmitic acid (C_15_H_31_COOH), and other fatty acids from C14 to C20. To calculate the amount of ZnO to technical StA in complex preparations, the assumption was made as for StA of 100% purity. Considering the difference in molecular weight between palmitic and stearic acid, a 3% excess of carboxylic acid vs. calculated molar ratio for 100% purity of StA should be expected. However, we decided to accept the possible slight excess of carboxylic acids in stoichiometric reactions.

**Figure 2:**
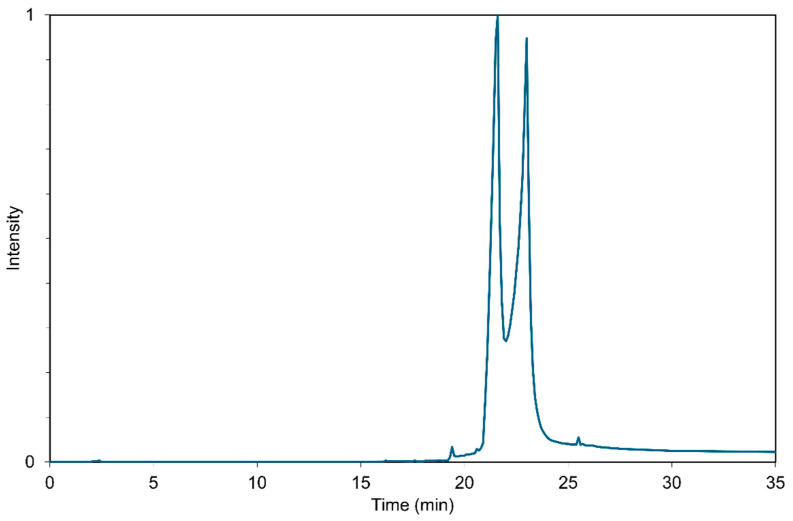
TD-GC-MS chromatogram of technical stearic acid used for synthesis reactions.

The TD-GC-MS chromatogram presented in Figure 2 confirmed the compliance of the material with the manufacturer’s declaration. The first peak (~21 min) corresponds to palmitic acids and the second (~23 min) to stearic acid. There are visible traces of shorter and longer carboxylic acids on the chromatogram as well.

### 2.3. Preparation of Rubber Compounds

Three series of rubber compounds, with compositions given in Table 2 and Table 3, were prepared and studied.

The first series, which consisted of the reference rubber compounds (Ref0, Ref1, and Ref2, respectively), was manufactured in a standard way using a two-roll mill (David Bridge & Co., Rochdale, UK). The reference rubber compounds contained standard ZnO or active ZnO and other components of the cross-linking system, i.e., sulfur, accelerators, and StA, added separately. During the preparation of the reference rubber compounds, the masterbatch was first plasticized for 3 min, and then ZnO, StA, and sulfur were added successively and mixed using the two-roll mill. The total time for preparing the rubber compound was 10 min. The mixing process was carried out at temperatures up to 50 °C, since in such conditions no chemical reaction between ZnO and stearic acid was expected before vulcanization.

The other two series of rubber compounds, which contained the previously prepared synthesis products obtained in the reaction of stearic acid, zinc oxide, and sulfur (S_8_), were prepared using a Brabender mixer. These materials contained synthesis products together with a rubber masterbatch without the ingredients used to prepare the synthesis products and accelerators included in the cross-linking system. The aim of this task was to homogeneously mix the melted ingredients together with the masterbatch before mixing them with the rest of the masterbatch and vulcanization accelerators, which would not be possible at the later stage without starting the vulcanization process. Initial Brabender chamber temperature was set at 115 °C, with a mixing speed of 45 revolutions per minute (rpm). After masterbatch dosing, the speed was kept at 45 rpm for 1 min before other ingredients were dosed. After performing complete dosing, the speed was changed to 70 rpm for 5 min and then to 90 rpm. The discharge of premixing was when the temperature of mixing rose to a minimum of 130 °C. After introducing the synthesis products into the masterbatch in the molten state using a Brabender mixer, vulcanization accelerators and sulfur were added successively using a two-roll mill. and the sheets of rubber compounds were prepared after 10 min of mixing.

To check the differences between synthesized materials and commercial ZnSt, additional rubber compounds described in Table 3 were prepared. Two additional rubber composites (Test18 and Test19, respectively) were also manufactured to check if a higher amount of StA is necessary for accelerators and can have some positive impact on vulcanizate properties.

**Figure 3:**
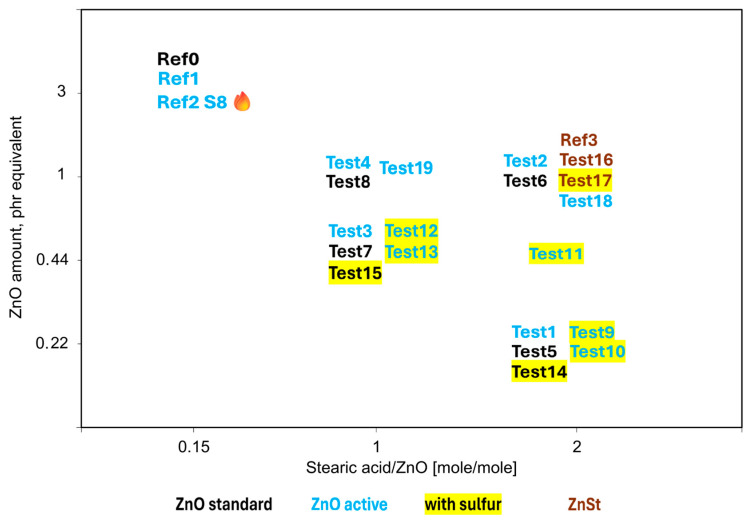
Drawing of the test concept for rubber compounds described in Table 2 and Table 3.

All rubber compounds and test conceptions are summarized in Figure 3 below.

### 2.4. Research Methods

Rheometric tests for rubber compounds were performed using Production Moving Die Rheometer MDR-P (Alpha Technologies, Hudson, OH, USA). The cure characteristics of EPDM compounds were studied at a temperature of 180 °C for the test time of 7 min.

Viscosity tests for rubber compounds were made using the MV 2000 Mooney viscometer (Alpha Technologies, Hudson, OH, USA) at a temperature of 125 °C according to the scorch test.

Samples of vulcanizates for compression set (CS) measurements and swelling tests were prepared using the Argenta AW 03 vulcanization press (Argenta, Kielce, Poland) with bottom and top heating plates and a temperature control system. Plates of vulcanizates with a thickness of approximately 2 mm were obtained at 180 °C and time of 10 min. There were five samples for each test prepared to minimize faults of measurements.

The equilibrium swelling tests were conducted for EPDM vulcanizates. Samples of rubber cut from 2 mm plates of vulcanizates were weighed on a laboratory scale and put in steel cans with xylene (mixture of isomers, purity 98%, Avantor Performance Materials S.A., Gliwice, Poland) for 48 h at a room temperature of approximately 23 °C. Then, the swollen samples were dried using a paper towel and secondly weighed. The equilibrium swelling (*Q_w_*) of the vulcanizates was calculated using Equation (1):

(1) $$Q_{w}=\frac{m_{1}-m_{0}}{m_{0}}\cdot 100\%,$$

where

*Q_w_*—equilibrium swelling (%),

*m*_1_—weight of the swollen sample (mg),

*m*_0_—weight of the sample before swelling (mg).

Compression set tests were performed using the standard procedure described in ISO 815-1:2019 method B [21]. The samples with a thickness of 6 mm were prepared from three layers of 2 mm plates. The height of the samples was measured, and then they were compressed by 25% for 22 h at 70 °C. After 2 h of conditioning at room temperature in a compressed state and 30 min of relaxation after removing the external stress, the height of the samples was secondly measured, and *CS* was calculated using Equation (2):

(2) $$CS=\frac{h_{0}-h_{2}}{h_{1}},$$

where

*h*_0_—initial thickness of the sample before test (mm),

*h*_1_—height of compression (mm),

*h*_2_—final thickness of the sample after test (mm).

The mechanical properties of vulcanizates were explored using the Zwick Roell 1435 (ZwickRoell, Ulm, Germany) universal testing machine. Tensile tests were carried out for four paddle-shaped samples with a thickness of approximately 1 mm and the width of the measuring section of 4 mm. All the samples were cut on press from vulcanizates cured at the temperature of 180 °C for 10 min. The crosshead speed during tensile tests was 500 mm/min.

Differential Scanning Calorimetry (DSC) tests were performed for the synthesized products employing the DSC1 analyzer (Mettler Toledo, Greifensee, Switzerland). Measurements were carried out at a temperature range from 25 °C to 200 °C, with a heating rate of 10 °C/min. The flow rate of nitrogen during the measurement was 80 mL/min. Based on DSC data, the expected heat of zinc stearate fusion was determined for synthesized products using Equations (3)–(8):

(3) $$mStA=\frac{m_{s}\cdot \;LStAs}{LStA},$$

where

*mStA*—mass of stearic acid in DSC sample (mg),

*m_s_*—mass of DSC sample (mg),

*LStAs*—DSC measured sample heat of melting about StA phase transition temperature (J/g),

*LStA*—DSC measured heat of melting for pure StA (J/g).

(4) $$m_{0}StA\;=\frac{m_{s}\;\cdot \;2\;\cdot \;MStA}{2\;\cdot MStA\;+\;a\;\cdot \;MZnO},$$

where

*m*_0_*StA*—initial mass of stearic acid in sample (mg),

*MStA*—molar mass of StA (g/mol),

*MZnO*—molar mass of ZnO (g/mol),

*a*—molar proportion of ZnO in recipe (Table 1).

(5) $$mRStA=m_{0}StA-mStA,$$

where

*mRStA*—reacted stearic acid during synthesis (mg).

(6) $$m_{y}ZnSt=\frac{mRStA\;\cdot \;MZnSt}{2\;\cdot \;MStA},$$

where

*m_y_ZnSt*—zinc stearate yielded (mg),

*MZnSt*—molar mass of ZnSt (g/mol).

(7) $$LeZnSt=\frac{m_{y}ZnSt\;\cdot \;LZnSt}{m_{s}},$$

where

*LeZnSt*—expected heat of melting of zinc stearate yielded (J/g),

*LZnSt*—expected heat of melting of commercial zinc stearate (J/g).

(8) $$\%y=\frac{mRStA}{m_{0}StA}\cdot 100\%$$

where

%*y*—relative effectiveness of stearic acid conversion to carboxylic acid salt for stoichiometry products (%).

Fourier Transform Infrared Spectroscopy (FTIR) measurements for synthesized products were carried out using a ThermoScientific Nicolet 6700 FT-IR spectrometer (Thermo Fisher Scientific, Waltham, MA, USA) equipped with OMNIC 3.2 software. The attenuated total reflectance (ATR) technique with a single diamond ATR crystal was used for the tests. FTIR absorbance spectra were recorded in the wavenumber range of 4000–400 cm^−1^ using 128 scans.

Scanning Electron Microscopy (SEM) was used to investigate the distribution of solid particles in the rubber matrix. SEM images of the vulcanizates were taken using an SEM LEO 1450 microscope (Carl Zeiss AG, Oberkochen, Germany). Before measurement, the vulcanizates were fractured after cooling in liquid nitrogen, and their surfaces were covered with a thick layer of carbon. SEM with Energy-Dispersive X-ray Spectroscopy (EDS) was used to study the composition of agglomerates. SEM/EDS studies were performed using a HITACHI S-4700 (Hitachi, Mannheim, Germany) SEM microscope.

Thermal Desorption–Gas Chromatography–Mass Spectrometry (TD-GC-MS) was used for stearic acid composition verification. The apparatus used for analysis was from Perkin-Elmer (Shelton, CT, USA). Tests were performed by desorption at 200 °C on TurboMatrix 650. The column used for chromatography was Clarus 680, with He gas flow 2 mL/min. For identification of materials, mass spectroscopy on Clarus 600 C was used in EI+ ionization mode.

## 3. Results and Discussion

### 3.1. Characterization of the Zinc Salts and Complexes

**Figure 4:**
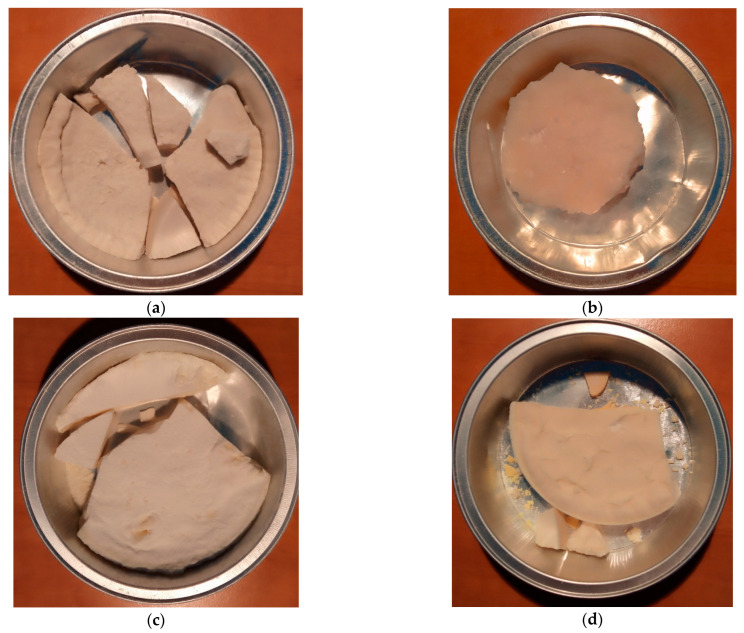
Images of synthesized products: (**a**) StZn_s_1; (**b**) StZn_a_1; (**c**) StZn_a_2; (**d**) StZnS_a_1_7.

While heating the components of the mixtures presented in Table 1, the synthesis reaction of ZnSt was observed, along with the release of water. In the case of active ZnO, this process was much more turbulent than for standard ZnO. In the case of samples with added sulfur, it was observed that the mixture delaminated due to the separation of melted sulfur in the lower part of the reaction flask. The prepared synthesis products were poured into aluminum containers and left to cool. The obtained products are presented in Figure 4.

**Figure 5:**
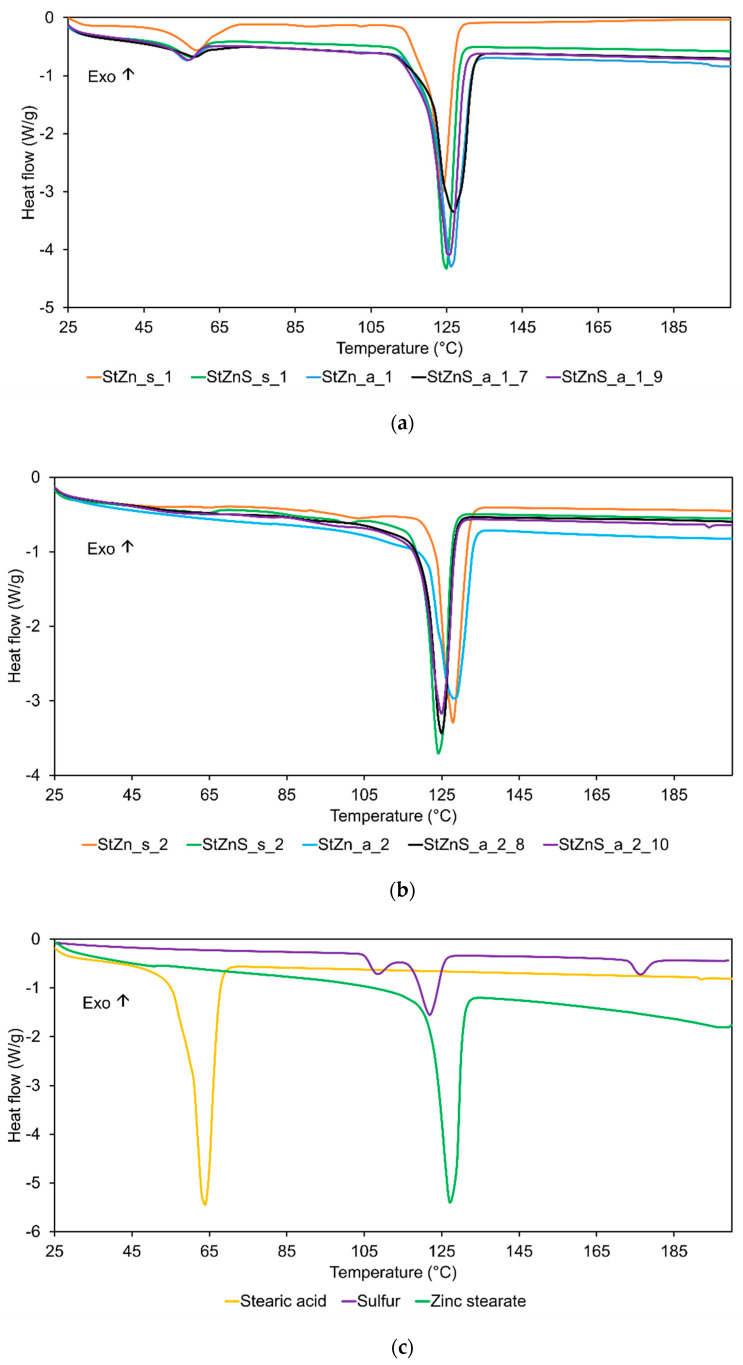
DSC results for synthesis products described in Table 1: (**a**) stoichiometric; (**b**) non-stoichiometric; (**c**) raw materials, i.e., commercial StA, S, and ZnSt.

The obtained synthesis products were subjected to DSC analysis in order to observe the differences in thermal transitions occurring during their heating and identify the synthesis products, as well as the presence of unreacted substrates. DSC curves for the synthesis products are presented in Figure 5a,b. Additionally, DSC measurements were conducted for technical stearic acid and pure sulfur (S8) (Figure 5c).

Analyzing the DSC curves presented in Figure 5a, it was observed that the products obtained from the mixture containing 2 moles of StA per 1 mole of ZnO showed a melting of residual carboxylic acid in the temperature range of 50–70 °C. The residual carboxylic acid content resulted from the composition of technical stearic acid, which was a blend of palmitic and stearic acid. As mentioned earlier, the average molecular weight of acid blends is lower than that of the pure stearic acid, which resulted in the excess of carboxylic acid in the prepared mixture. The second intensive endothermic peak on the DSC curve in the temperature range of 110–130 °C was attributed to the melting of the products resulting from the reaction between StA and ZnO. However, for non-stoichiometric products, no melting peak for the residual carboxylic acid was observed, but only the sharp endothermic peak for melting of the synthesis products (Figure 5b).

On the other hand, for stoichiometric products, the DSC curves did not change significantly regardless of whether a standard or active ZnO was used. The addition of sulfur had no significant effect on the melting peak of the main synthesis product. Some residual endothermic peaks were observed on DSC curves at a temperature of 105 °C for synthesis products containing sulfur, which resulted from the allotropic transformation of sulfur.

From DSC measurements, the heat of the melting and peak temperatures for synthesized products and raw materials were determined and summarized in Table 4.

The heat of melting determined for the zinc stearate sample was 153.3 J/g, lower than reported in the literature. Konkoly-Thege et al. [22] reported it as 103 kJ/mol, which gives 162.9 J/g. These researchers also determined the heat of melting for zinc palmitate as 93 kJ/mol, which gives 161.4 J/g. The melting temperatures were reported as 130 °C for zinc stearate and 134 °C for zinc palmitate, also a bit higher than the peak temperatures determined from DSC data in our study. The significantly higher heat of melting for residual carboxylic acid in the case of standard ZnO (sample StZn_s_1) compared to active ZnO (sample StZn_a_1) revealed that the standard ZnO was not fully converted during the reaction. It is also visible that the heat of melting of the carboxylic acid salt for the standard ZnO (sample StZn_s_1) was significantly lower than that for the active ZnO (sample StZn_a_1). A similar dependence was observed in the case of ZnO excess; the heat of melting for zinc salt produced from standard ZnO (sample StZn_s_2) was significantly lower than that for active ZnO (sample StZn_a_2).

Based on the DSC data, the expected heat of zinc stearate fusion was determined for synthesized products without sulfur and listed in Table 5. Mixtures with sulfur were excluded from estimations because part of the sulfur from those mixtures was not incorporated into the synthesized products and stayed on the bottom of the aluminum containers. On the other hand, the part of sulfur which was incorporated into the synthesis product had a similar melting point to ZnSt.

The relative effectiveness of stearic acid conversion to carboxylic acid salt for stoichiometry products was 85% for standard ZnO and 93% for active ZnO (excess of acid), respectively. The higher effectiveness of the reaction for excess of ZnO (samples StZn_s_2 and StZn_a_2, respectively) can suggest that the reaction efficiency was linked to the size of the ZnO particles. The presence of a carboxylic acid melting peak suggests that there is no formation of non-stoichiometric ZnSt containing more carboxylic acid. However, this observation does not eliminate the possibility that a non-stoichiometric complex with an excess of Zn has been created. The heat of the melting of StA is higher than would be expected from a 3% excess of StA, which means that not all the ZnO has reacted. ZnO was dissolved by the carboxylic acid gradually. Thus, there is a residue of both carboxylic acid and ZnO or its impurities in the preparation. Based on the calculated results of reacted StA from DSC measurements and its 3% excess, non-reacted or complexed ZnO is about 12% for StZn_s_1 (standard ZnO) and 4% for StZn_a_1 (active ZnO).

The heats of melting for synthesized products differed from those determined theoretically based on DSC-measured stearic acid remains. For standard ZnO, they were lower than expected, but for active ZnO the results were opposite. The heats of melting determined through DSC for products from non-stoichiometric mixtures were significantly lower than for the stoichiometric one. On the other hand, the counted expected heats of melting were quite similar. All those differences could result from some non-stoichiometric zinc stearate particles formed as a binuclear bidentate or crystal structure of products yielded during synthesis. Zinc stearate can exhibit different crystal structures depending on the synthesis conditions, with two main types of geometry observed in zinc soaps, i.e., a highly symmetrical packing for long-chain saturated soaps and an alternating packing for zinc soaps with shorter, unsaturated, or dicarboxylic chains [23]. The method used to synthesize ZnSt can influence its crystal structure. For example, precipitation and fusion processes can yield ZnSt with slightly different melting points and crystal morphologies. The phase transition in ZnSt is not just a simple melting of the alkyl chains [24]. It also involves changes in the coordination of the stearate ions to the zinc ions, potentially forming ionic clusters in the liquid phase. In ZnSt, each zinc ion is coordinated to two stearate anions. This bidentate coordination is believed to contribute to the relatively high melting point and enthalpy of fusion compared to simple alkanes. At the temperature of the industrial vulcanization process, i.e., around 180 °C, the crystal structure of the yielded ZnSt should not have an impact on the vulcanization course and efficiency because those products should be in a liquid state.

**Figure 6:**
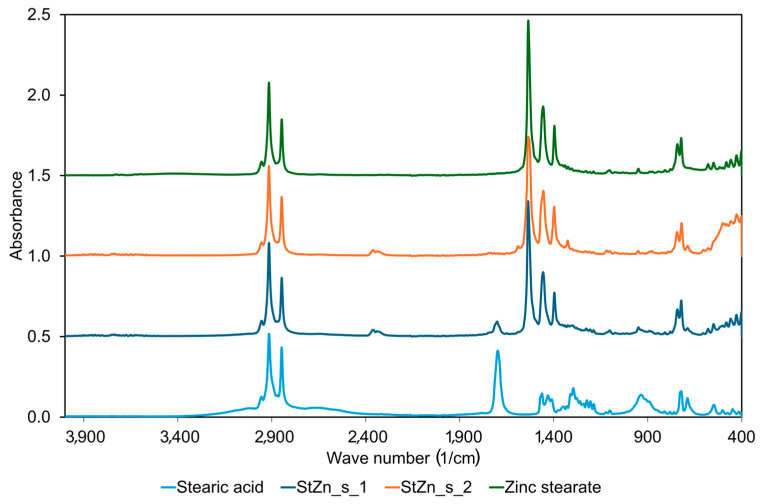
FTIR spectra for commercial StA, commercial ZnSt, and product of synthesis, i.e., StZn_s_1 and StZn_s_2, respectively.

The transitions of substrates into zinc salts in the reactive mixtures were checked using FTIR Spectroscopy. The FTIR spectra of StA used for the synthesis and commercial ZnSt were collected to compare them with those obtained for the reaction products prepared in stoichiometric (StZn_s_1) and non-stoichiometric (StZn_s_2) conditions (Figure 6). Regarding the FTIR spectrum for StA, the absorption peaks at about 2915 and 2849 cm^−1^ in the high-frequency region were attributed to −CH_2_− asymmetric and symmetric stretching vibrations, respectively. They were accompanied by stretching vibrations of the −CH_2_- band at a wave number of approximately 1472 cm^−1^ [25]. Furthermore, the strong and sharp peak at approximately 1700–1710 cm^−1^, characteristic of the C=O stretching vibrations in the carboxylic acid group, and peaks at 1300–1420 cm^−1^, characteristic of the O-H bending vibrations of the hydroxyl group in carboxylic acids, were observed. In addition, peaks corresponding to −C–O stretching vibrations in −COOH were identified in the wave number range of 930–1100 cm^−1^ [26]. It should be noticed that, in both spectra for synthesized products, the band at a wave number of approximately 1700 cm^−1^ characteristic for the double bond between carbon and oxygen in carboxylic acids, i.e., C=O band, changed to a sharp peak at approximately 1535 cm^−1^, which is typical for asymmetric stretching vibrations of −COO^−^ in carboxylate salts, accompanied by sharp peaks corresponding to symmetric stretching vibrations of −COO^−^ at approximately 1400–1450 cm^−1^ [26]. This confirmed that a reaction took place between ZnO and stearic acid, and a carboxylic acid salt was formed. On the spectrum of StZn_s_1, it is also visible that a part of StA remained unreacted, since the small peak at approximately 1700–1710 cm^−1^, characteristic of the C=O stretching vibrations in the carboxylic acid group, is still present. On the other hand, in the FTIR spectrum of StZn_s_2, the presence of non-reacted ZnO can be concluded, since the intensity of the peak at a wave number of approximately 400–600 cm^−1^, which is typical for Zn–O stretching vibrations [27], is higher compared to the FTIR spectrum of the product synthesized in stoichiometric conditions, i.e., StZn_s_1 or pure ZnSt. The intensity of the bands in this wave number range is higher than for the product synthesized in stoichiometric conditions, even though this is already the limit of the measuring range of the device. Due to fact that, in this range of wave numbers, the absorbance is imposed with a ZnSt answer, i.e., Zn-O stretching, which confirms metal–oxygen bonding, it is difficult to determine the presence of unreacted ZnO for ZnSt_s_1. There is no difference in the spectra of ZnSt_s_1 and ZnSt_s_2 other than the lack of bands at 1700 cm^−1^ (typical for C=O group in carboxylic acids) and approximately 420 cm^−1^ (typical for Zn-O vibrations) in the spectrum of ZnSt_s_2. This observation supports the findings concluded based on DSC results that only carboxylic acids salts were formed without non-stoichiometric complexes.

### 3.2. Characterization of the EPDM Composites Containing the Synthesized Zinc Salts and Complexes

Having characterized the products of the performed synthesis, we used them to prepare rubber compounds. Then, the effects of standard ZnO, active ZnO, and the synthesized zinc salts and complexes on the rheometric properties of EPDM compounds were explored. The results are presented in Table 6.

Analysis of the measurement results performed using the MDR and Mooney viscometer (Table 6) showed that, for the reference rubber compounds, the application of active ZnO (Ref1) increased the torque increment during the rheometric test (MH-ML), which is an indirect measure of the cross-linking degree of the rubber compound, whereas a slightly longer optimal vulcanization time t_90_ (time to reach 90% of maximum torque during rheometric test) compared to standard ZnO (Ref0) was achieved. This confirmed the higher activity of active ZnO compared to standard ZnO, which was due to the larger specific surface area of active ZnO, resulting in a larger contact area between the components of the cross-linking system during vulcanization. Applying materials synthesized using active ZnO (Test1 to 4) allowed us to achieve the same trend compared with the materials obtained based on standard ZnO (Test5 to 8), but the differences were not so significant as in the case of the rubber compounds containing raw zinc oxides. Rubber compounds with a 3-times reduced quantity of ZnO showed both a higher MH–ML and longer t_90_ for active ZnO vs. standard ZnO (Test2 vs. 6 and Test4 vs. 8, respectively), which additionally confirmed the greater activity in vulcanization of both active ZnO and the synthesis product obtained using it. For rubber compounds containing a zinc amount equivalent to stearic acid in the reference rubber compound, the differences in MDR and Mooney test results compared to the standard and active ZnO were not so significant (Test1 vs. 5 and Test3 vs. 7). The rubber compounds containing materials synthesized with sulfur exhibited MDR and Mooney values similar to rubber compounds containing the synthesis products obtained using only StA and ZnO with the same ZnO equivalent (Test1, 9, 10 vs. 3, 12, 13). Adding sulfur before ZnO during the synthesis resulted in the decrease in t_90_ only for rubber compounds containing materials obtained in stoichiometric conditions (Test9 and 10, respectively). This indicated the lack of formation of sulfur complexes with ZnO and StA during the reaction—only sulfur melting and changes in its crystalline form took place during the mixing of the ingredients in the reaction flask. Applying commercial laboratory grade ZnSt (Ref3, Test16 and 17) resulted in torque values on similar levels as rubber compounds containing materials synthesized from standard ZnO, but with a significantly longer t_90_. The differences in the rheometric data obtained for Ref3 and premixed in the Brabender mixer rubber compounds (Test16 and Test17) revealed that mixing in the molted state, aiming to obtain a perfect dispersion of the curatives, improved the torque increment MH-ML and decreased the cure time, thus having a beneficial effect on the effectiveness of vulcanization. Additional amounts of stearic acid, which can influence the activity of accelerators, significantly reduced the torque increment MH-ML, deteriorating the cross-linking degree of the elastomer and slightly decreasing the t_90_, thus improving the rate of vulcanization (Test2 vs. 18 and Test4 vs. 19).

Having investigated the influence of standard and active ZnO, as well as their salts or complexes, with stearic acid on the vulcanization parameters, we then examined their impact on the functional properties of the EPDM vulcanizates, i.e., the compression set (CS), which is crucial for sealing applications. The results are presented in Table 7.

As it is widely known, the kind of ZnO and its quantity have a significant impact on vulcanizate properties. Analyzing the data collected in Table 7, rubber composites with active ZnO exhibited better properties, i.e., a lower CS and equilibrium swelling, compared to standard ZnO. However, when the synthesized materials based on these types of ZnO were applied, the differences between the obtained results were not so significant as in the case of reference composites with both types of ZnO. The CS and equilibrium swelling values decreased for EPDM vulcanizates with active ZnO (Ref1 and Ref2) compared to standard ZnO (Ref0). The lower equilibrium swelling allowed us to conclude that the active ZnO ensured that the vulcanizates had a higher cross-linking density compared to the standard ZnO. This correlates well with the results of rheometric tests. Ref3 with zinc stearate exhibited the best functional properties, i.e., the lowest CS, among all references, despite the fact that its zinc equivalent was three times lower than for other reference rubber compounds. A better dispersion of zinc and sulfur for rubber compounds premixed in the Brabender probably led to an improvement in the vulcanizate properties despite the zinc content reduction. This trend was observed for both kinds of ZnO and for zinc stearate.

The addition of stoichiometric zinc salt in an amount that was 3 times lower than in the reference (Test2) resulted in a significant improvement in CS (52.9% vs. 60.9%) obtained for Ref1, with a reduction in equilibrium swelling (74.7% vs. 85.6%, respectively), and, consequently, an increase in the cross-linking density of the vulcanizate. A further decrease in the zinc salt amount to a 13.6-times (Test1) lower equivalent of ZnO resulted in a CS on a level similar to vulcanization with standard ZnO, i.e., 73.7% vs. 72.5% (Ref0), whereas the equilibrium swelling increased significantly to 103.9% due to the decrease in the cross-linking density of the vulcanizate. Stoichiometric zinc salts produced from standard ZnO resulted in only a slightly higher CS of vulcanizates compared to zinc salts produced from active ZnO (Test5 vs. Test1 and Test6 vs. Test2, respectively), which was also in line with the increase in equilibrium swelling. Slight differences (on the level of measurement uncertainty) between the results obtained for vulcanizates containing zinc salts synthesized from active ZnO and standard ZnO suggest that only the salt amount is important for the properties of vulcanizates, not the kind of ZnO used for the synthesis.

Non-stoichiometric products of synthesis, which contained almost half of the unreacted ZnO and half of the ZnSt, resulted in a slightly lower CS of vulcanizates compared to stoichiometric ZnSt only for Test3 vs. Test1, but for Test4 vs. Test2 the relationship was opposite. Those changes are in line with the equilibrium swelling results (lower equilibrium swelling and consequently higher cross-linking density accompanied by lower CS of vulcanizates). Hence, in order to obtain the proper CS of vulcanizates, the more important factor is not the type of vulcanization activator, but the cross-linking density of the vulcanizate, which can be obtained by using this particular activator. For vulcanizates containing the products synthesized from standard ZnO, the effects of additional amounts of ZnO on the CS and equilibrium swelling of vulcanizates have the same trend. Applying the mixtures containing ZnO, StA, and sulfur resulted in vulcanizates with CS and swelling results slightly lower for a very low ZnO equivalent (Test9 and 10 vs. Test5, 13.6-times lower zinc amount than in Ref1) and slightly higher for a low ZnO equivalent (Test12 and 13 vs. Test3, 6.7-times lower zinc amount than in Ref1). This suggests that sulfur did not form any complex with ZnO and StA during mixing; the obtained differences in CS of vulcanizates can be only due to the impact of ZnSt and sulfur content. A comparison of the CS and swelling results of vulcanizates for Test13 and 12, which contained the products obtained when sulfur was added before or after ZnO addition during the synthesis, showed no significant differences considering the measurement uncertainty. This supports the earlier statement that there was no formation of sulfur complex during the reaction of sulfur, ZnO, and StA. A comparison of the results obtained for vulcanizates containing synthesized products using standard ZnO vs. active ZnO for stoichiometric salts allowed us to observe that there were no significant differences between CS and swelling values for Test14 (standard ZnO) and Test9 (active ZnO). CS values for both vulcanizates were approximately 70.4%, and the equilibrium swelling of these vulcanizates was also similar, which indicates a similar cross-linking density. This further confirmed that only the amount of salt is important. A similar relationship was observed for Test15 vs. 12, i.e., zinc salts obtained from standard ZnO vs. active ZnO in non-stoichiometric conditions. An analysis of the obtained results allowed us to conclude that the excess of StA combined with ZnSt had a very important effect on the CS of the vulcanizates. For vulcanizates with ZnSt, i.e., Ref3, Test16, and Test17, CS was reduced to 41.5–44.5% from approximately 60.9% (Ref1—with active ZnO). This is an even better result than for Test2 (stoichiometric synthesis product, 1 phr of active ZnO), which exhibited a CS of approximately 52.9%. It proved that raw ZnSt ensured better curing conditions than a mix of other zinc salts, with ZnSt received in the product synthesized from commercial StA. The equilibrium swelling also decreased significantly, indicating a marked increase in the cross-linking density of the vulcanizates, which probably contributed to the decrease in CS. This confirmed that a better mixing of ZnSt can improve the availability of zinc ions during cross-linking reactions. A comparison of the results obtained for Test18 (1 phr of stoichiometric ZnSt from active ZnO) vs. Test2 (with addition of StA) showed no improvement in the CS; however, the equilibrium swelling increased, indicating a deterioration of the cross-linking density of the vulcanizate. This suggests that a small excess of StA can be beneficial for the activation of accelerators. On the contrary, a comparison of the results for Test19 (1 phr of non-stoichiometric ZnSt from active ZnO) vs. Test4 (with addition of StA) revealed an improvement in the CS, while Test4 did not contain any excess of StA. Thus, it was concluded that StA acted not only as a ZnSt precursor, but also as an activator of accelerators.

Most importantly, the performed studies proved that adding materials synthesized in the reaction of ZnO, StA, and sulfur could help to reduce the content of zinc in rubber composites, while keeping a good level of both important manufacturing parameters, like the viscosity and cure time of rubber compounds, and also functional properties, i.e., the CS or cross-linking density, of the vulcanizates.

In the next step of the study, the tensile properties of the vulcanizates were examined. The results are presented in Table 8.

**Figure 7:**
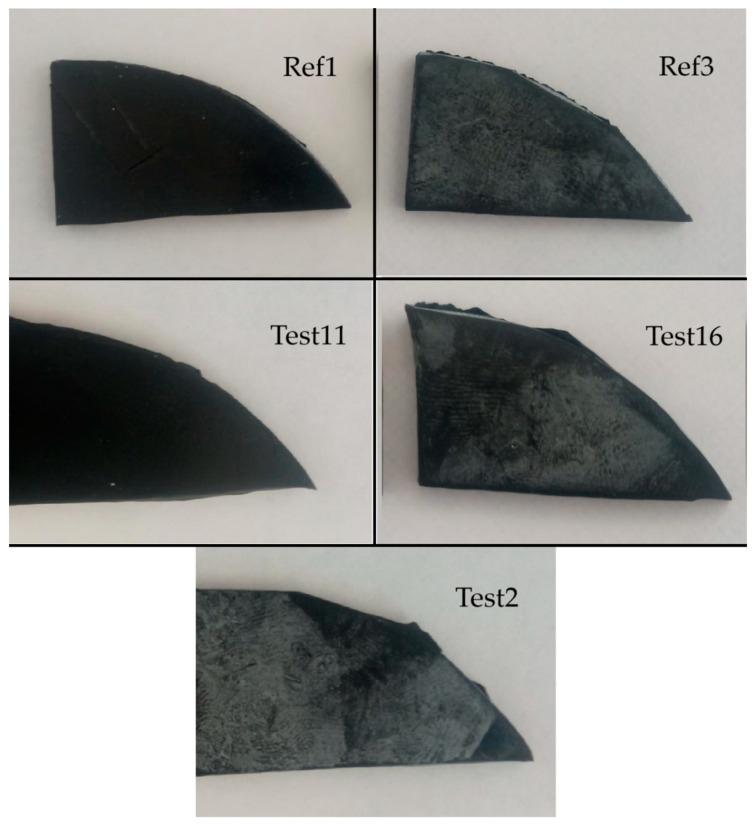
Blooming of vulcanizates described in Table 2 and Table 3.

The reference vulcanizate Ref0 with standard ZnO exhibited the lowest tensile strength (TS) and elongation at break (E_b_). It should be noted that the E_b_ of this vulcanizate was approximately 120%, while for most other vulcanizates the E_b_ was above 400%. This indicates a very low elasticity of this vulcanizate compared to the others, which may be caused by its significantly higher degree of cross-linking. The vulcanizate with active ZnO (Ref1) showed twice the TS compared to that with standard ZnO, and a significantly better elasticity (E_b_ of approximately 500%). The vulcanizate with ZnSt (Ref3) also showed better tensile parameters, i.e., TS and E_b_, compared to that with standard ZnO, despite having a 3-fold lower zinc equivalent. Most importantly, vulcanizates containing the synthesized zinc complexes exhibited a significantly higher TS and E_b_ than vulcanizates with standard (Ref0) or active ZnO (Ref1). Surprisingly, vulcanizates with a 13.6-times lower zinc amount (Test9 and 14) compared to the reference samples showed much better tensile properties despite a significantly higher equilibrium swelling and, consequently, lower cross-linking degree. This allowed us to assume that the ZnO-containing vulcanizates were slightly over-cross-linked compared to those containing synthesis products, which caused a deterioration of their TS and an increase in their brittleness. On the other hand, a high amount of StA in rubber compounds creates a huge issue to solve, i.e., the whitening of vulcanizates (blooming) [28]. With time, additives like the zinc salts of fatty acids migrate on the surface of vulcanizates, causing visual aspects and influencing the adhesion [29]. A higher content of StA in the recipe of rubber compounds results in a higher amount of ZnSt in the vulcanizates. In Figure 7 the problem is presented for the vulcanizates containing active ZnO with the standard amount of StA (Ref1), the reference with ZnSt (Ref3), Test16 with an StA equivalent about 5-times higher than in Ref1, Test2 with StA equivalent 6.7-times higher than in Ref1, and Test11 with an StA equivalent 2-times higher than in Ref1. Vulcanizates with a 2-times higher content of StA compared to Ref1, i.e., Test11, did not show any visual blooming aspects. The amount of created ZnSt in the reaction of StA with ZnO during the preparation of the rubber compound and the curing process should also be twice as high accordingly. The increasing of the ZnSt amount in the vulcanizate by a higher amount of StA led to an unacceptable migration on the vulcanizate surface (Ref3, Test2, and Test16). For rubber composites containing synthesized materials, the reduction of zinc in the amount that did not impact their functional properties was not enough. In turn, a further reduction in the zinc amount to keep the equivalent StA as in the standard rubber compound (Ref1) or not creating blooming issues (Test11) did not guarantee keeping the demanded properties of the vulcanizates.

**Figure 8:**
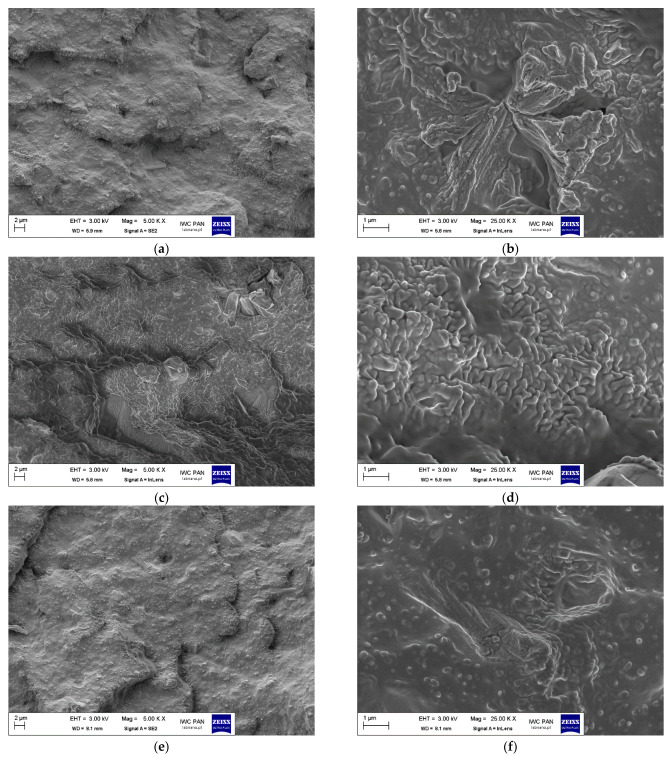

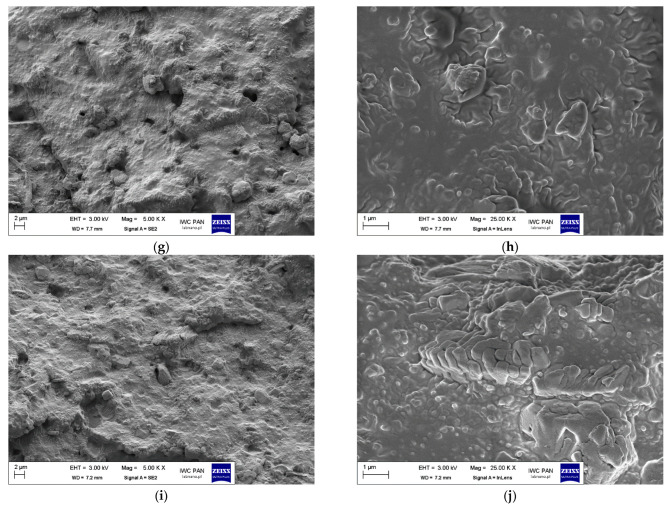
SEM images of vulcanizate fractures: (**a**,**b**) Ref1; (**c**,**d**) Test2; (**e**,**f**) Test11; (**g**,**h**) Test16; (**i**,**j**) Test18.

Parts of vulcanizates were examined using SEM to check whether their composition or the method of introducing the crucial cross-linking components (ZnO, StA, sulfur, ZnSt) separately or as products of the reaction influences their dispersion in the elastomer matrix. SEM images of the vulcanizate fractures are presented in Figure 8.

In the case of the reference vulcanizate with active ZnO, the overall dispersion of the components was relatively homogeneous (Figure 8a), although some agglomerates with a complex morphology and a size of several micrometers, representing clusters of primary particles less than 1 micrometer in size, also occurred (Figure 8b). These agglomerates were quite well wetted by the elastomer; most of the agglomerate visible in the photo was surrounded by an elastomer film. In the case of the vulcanizate containing stoichiometric ZnSt synthesized from active ZnO, the SEM image (Figure 8c) shows areas of both uniform particle dispersion and micrometric agglomerates. These agglomerates resulted from the agglomeration of particles smaller than 1 micrometer and demonstrated a better wettability by the elastomer compared to the agglomerates present in the SEM image of the vulcanizate containing active ZnO (Figure 8d). The elastomer film is clearly visible, penetrating between the agglomerate particles and surrounding them on all sides. The use of the product obtained by adding sulfur in the synthesis process of zinc salts/complexes did not have a significant effect on the overall dispersion of the mixture components (Figure 8e), although it seems that the wettability of the agglomerate visible in the SEM image (Figure 8f) is slightly worse in relation to the vulcanizate containing the synthesis product without sulfur. In the case of the vulcanizate containing commercial ZnSt, the dispersion of the components can be considered the most homogeneous (Figure 8g). The wettability of the particles and their aggregates by the elastomer is very good, as all of them are coated with an elastomer film and embedded in it (Figure 8h). Overall, this vulcanizate exhibited one of the lowest CSs and highest cross-linking densities. In the case of the vulcanizate containing stoichiometric ZnSt synthesized from active ZnO with an additional amount of StA, the overall dispersion of the components in the elastomer matrix is quite good (Figure 8i), but several micrometer-sized agglomerates of varying morphology are also visible, representing clusters of relatively large primary particles with a fairly regular platelet or block shape (Figure 8j). These agglomerates demonstrate a low wettability by the elastomer.

**Figure 9:**
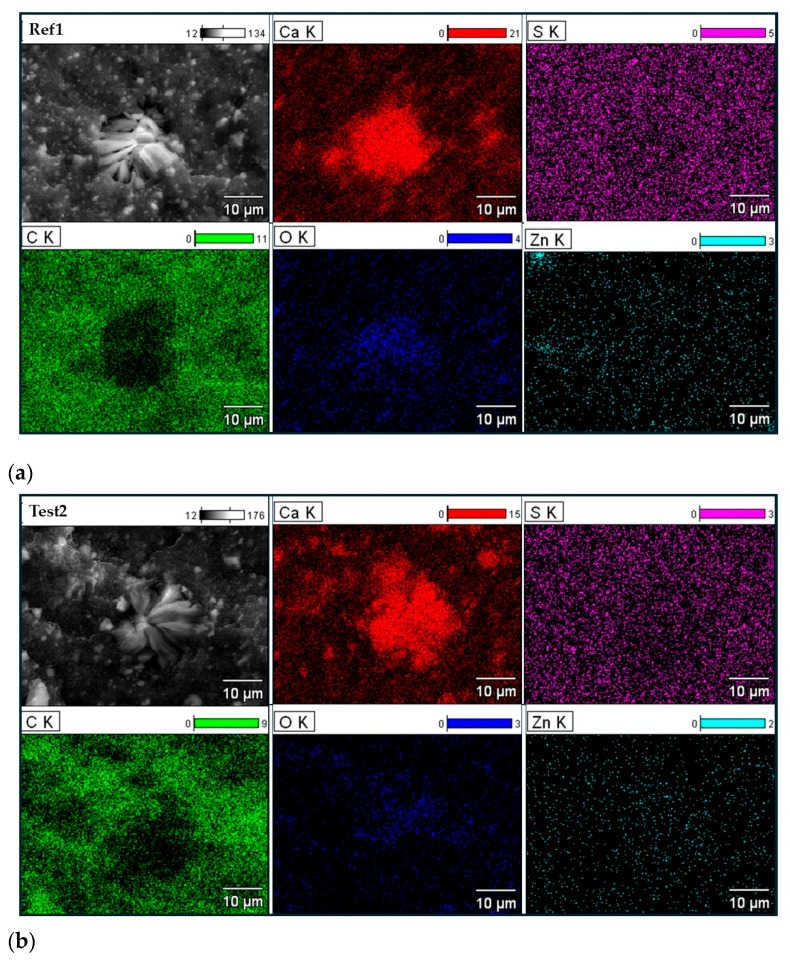

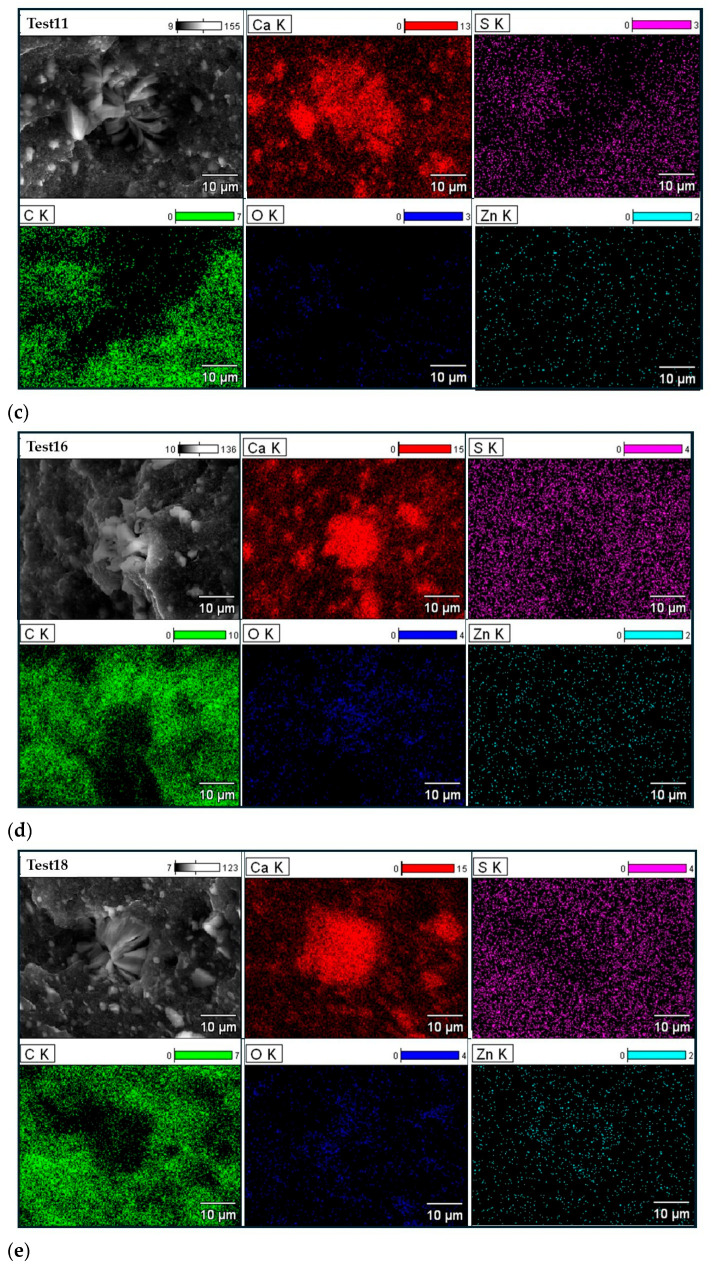
SEM images of vulcanizate fractures with EDS mapping for Ca, S, C, O, and Zn elements: (**a**) Ref1; (**b**) Test2; (**c**) Test11; (**d**) Test16; (**e**) Test18. Analysis of ESD mapping revealed that the agglomerates present in SEM images of the vulcanizates resulted from the agglomeration of calcium compounds present in the masterbatch composition, e.g., CaCO_3_. On the other hand, EDS analysis showed that components of the curing system, i.e., sulfur and zinc compounds, were homogeneously dispersed in the elastomer matrix, regardless of whether ZnO or the synthesized products obtained in the reaction of ZnO and StA under stoichiometric or non-stoichiometric conditions were used as the vulcanization activator. Hence, it can be concluded that the composition of the elastomer composite, i.e., the type and amount of the activator, did not significantly influence the general dispersion of the rubber composite components.

To identify the composition of the agglomerates visible in the above SEM images (Figure 8), SEM with EDS analysis was employed. The results in the form of EDS maps are presented in Figure 9.

## 4. Conclusions

In this research, zinc complexes with different molar ratios of ZnO and StA with or without the presence of sulfur were synthesized and studied as potential vulcanization activators for EPDM compounds. The vulcanization parameters and properties of the rubber composites containing synthesized materials were compared with those containing commercial ZnSt to verify the hypothesis on the key role in sulfur vulcanization played by non-stoichiometric zinc complexes.

DSC results confirmed that the reaction between ZnO and StA resulted in the formation of ZnSt. Complexes with different molar ratios of ZnO and StA contained the amount of ZnSt proportional to amount of StA used in the synthesis. In addition, an FTIR study of reaction products did not reveal any absorption bands different from those characteristic for StA and ZnSt. Active ZnO exhibited a higher reactivity in the formation of ZnSt than the standard ZnO, due to the higher specific surface area and lower size of particles that are consumed during the reaction. The heats of melting determined through DSC for the products from non-stoichiometric mixtures were significantly lower than for the stoichiometric ones. These differences could result from the difference in the crystallization process depending on the reaction conditions, since ZnSt particles can form binuclear bidentate or a crystal structure that differs in the heat of melting.

The stoichiometric salt of ZnO and StA was the most effective in terms of vulcanization parameters and the compression set of rubber composites. Non-stoichiometric complexes were less effective, proportionally to their content. However, they can also be a potential alternative to traditional ZnO and StA systems in the vulcanization of EPDM composites. The comparison with stoichiometric ZnSt revealed that the effect on the vulcanization state and compression set of rubber composites is mostly driven by stoichiometric salt. In case of non-stoichiometric complexes, the influence on the vulcanization parameters, cross-linking density, and compression set was proportional to the content of stoichiometric salt. Moreover, the amount of salt was more important than the type of ZnO used during the synthesis. Th excess of StA can improve the availability of zinc during cross-linking reactions, leading to improved properties of the vulcanizates. The addition of sulfur to zinc complexes did not significantly affect the vulcanization and properties of the EPDM composites, which disproved the hypothesis that sulfur formed complexes with StA and ZnO.

## Figures and Tables

**Table 1:** Composition of the mixtures of ZnO with StA and/or S in moles of each ingredient.

No.	Sample Symbol	Stearic Acid (mole)	Zinc Oxide ^1^ (mole)	Sulfur ^2^ (mole)
1	StZn_s_1	2	1	-
2	StZn_s_2	2	2	-
3	StZnS_s_1	2	1	8
4	StZnS_s_2	2	2	8
5	StZn_a_1	2	1 A	-
6	StZn_a_2	2	2 A	-
7	StZnS_a_1_7	2	1 A	8
8	StZnS_a_2_8	2	2 A	8
9	StZnS_a_1_9	2	1 A	8
10	StZnS_a_2_10	2	2 A	8

^1^ The ZnO used for the synthesis was in two variants: standard ZnO and active ZnO with increased specific surface area, marked with the letter A in the table. ^2^ Sulfur in samples No. 3, 4, 7, and 8 was added after reaction between ZnO and StA. Sulfur in samples No. 9 and 10 was added to StA before ZnO.

**Table 2:** Compositions of rubber compounds containing synthesized products described in Table 1.

Rubber Compound	Description	ZnO phr Equivalent	StA/Zn [mole/mole]
Ref0	Reference compound with standard ZnO.	3	0.15
Ref1	Reference compound with active ZnO.	3	0.15
Ref2	Reference compound with active ZnO and sulfur thermally transformed (200 °C/5 min) in masterbatch before adding accelerator set—experiment following the previous study [13].	3	0.15
Test1	Rubber compound with StZn_a_1. Targeted to stoichiometric ZnSt. Amount of StZn_a_1 equivalent to StA concentration in reference. A total of 13.6 times less Zn than the reference.	0.22	2
Test2	Rubber compound with StZn_a_1. Targeted to stoichiometric ZnSt. Amount of StZn_a_1 to obtain Zn equivalent 3 times less than the reference.	1	2
Test3	Rubber compound with StZn_a_2. Targeted to ZnSt complex. Amount of StZn_a_2 equivalent to stearic acid concentration in reference. A total of 6.7 times less Zn than the reference.	0.44	1
Test4	Rubber compound with StZn_a_2. Targeted to ZnSt complex. Amount of StZn_a_2 to obtain Zn equivalent 3 times less than the reference.	1	1
Test5	Rubber compound with StZn_s_1. Targeted to stoichiometric ZnSt. Amount of StZn_s_1 equivalent to StA concentration in reference. A total of 13.6 times less Zn than the reference.	0.22	2
Test6	Rubber compound with StZn_s_1. Targeted to stoichiometric ZnSt. Amount of StZn_s_1 to obtain Zn equivalent 3 times less than the reference.	1	2
Test7	Rubber compound with StZn_s_2. Targeted to ZnSt complex. Amount of StZn_s_2 equivalent to StA concentration in reference. A total of 6.7 times less Zn than the reference.	0.44	1
Test8	Rubber compound with StZn_s_2. Targeted to ZnSt complex. Amount of StZn_s_2 to obtain Zn equivalent 3 times less than in reference.	1	1
Test9	Rubber compound with StZnS_a_1_7. Targeted to ZnSt/sulfur complex. Sulfur in amount equivalent to S_8_ per one Zn, added at the end. Amount of StZnS_a_1_7 equivalent to StA concentration in reference. A total of 13.6 times less Zn than the reference. ^1^	0.22	2
Test10	Rubber compound with StZnS_a_1_9. Targeted to ZnSt/sulfur complex. Sulfur in amount equivalent to S_8_ per one Zn, added before ZnO. Amount of StZnS_a_1_9 equivalent to StA concentration in reference. A total of 13.6 times less Zn than the reference. ^1^	0.22	2
Test11	Rubber compound with StZnS_a_1_7. Targeted to ZnSt/sulfur complex. Sulfur in amount equivalent to S_8_ per one Zn, added at the end. Amount of StZnS_a_1_7 equivalent to double StA concentration in reference. A total of 6.7 times less Zn than the reference. ^1^	0.44	2
Test12	Rubber compound with StZnS_a_2_8. Targeted to ZnSt/sulfur complex. Sulfur in amount equivalent to S_8_ per one Zn, added at the end. Amount of StZnS_a_2_8 equivalent to StA concentration in reference. A total of 6.7 times less Zn than the reference. ^1^	0.44	1
Test13	Rubber compound with StZnS_a_2_10. Targeted to ZnSt/sulfur complex. Sulfur in amount equivalent to S_8_ per one Zn, added before ZnO. Amount of StZnS_a_2_10 equivalent to StA concentration in reference. A total of 6.7 times less Zn than the reference. ^1^	0.44	1
Test14	Rubber compound with StZnS_s_1. Targeted to ZnSt/sulfur complex. Sulfur in amount equivalent to S_8_ per one Zn, added at the end. Amount of StZnS_s_1 equivalent to StA concentration in reference. A total of 13.6 times less Zn than the reference. ^1^	0.22	2
Test15	Rubber compound with StZnS_s_2. Targeted to ZnSt/sulfur complex. Sulfur in amount equivalent to S_8_ per one Zn, added at the end. Amount of StZnS_s_2 equivalent to stearic acid concentration in reference. A total of 6.7 times less Zn than the reference. ^1^	0.44	1

^1^ NOTE: sulfur did not completely react with ZnSt during mixing in the reactive flask—the fraction of sulfur in the complex was lower than assumed; that is why a regular amount of sulfur was added. This can generate some excess of sulfur in the rubber compound.

**Table 3:** Composition of rubber compounds containing commercial ZnSt and synthesized products described in Table 1.

Rubber Compound	Description	ZnO phr Equivalent	StA/Zn [mole/mole]
Ref3	Reference compound with commercial ZnSt and 1 phr of StA. Amount of ZnSt to obtain Zn equivalent 3 times less than Ref1 (Table 2).	1	2.28
Test16	Rubber compound with commercial ZnSt and 1 phr of StA premixed in Brabender with masterbatch. Amount of ZnSt to obtain Zn equivalent like in Ref3.	1	2.28
Test17	Rubber compound with sulfur, ZnSt, and 1 phr of StA premixed in Brabender with masterbatch. Amount of ZnSt to obtain Zn equivalent like in Ref3.	1	2.28
Test18	Rubber compound with StZn_a_1. Targeted to stoichiometric ZnSt. Amount of StZn_a_1 to obtain Zn equivalent 3 times less than the reference. Composition like Test2 (Table 2) but added 0.5 phr of stearic acid. Similar StA/Zn ratio like in Ref3.	1	2.13
Test19	Rubber compound with StZn_a_2. Targeted to ZnSt complex. Amount of StZn_a_2 to obtain Zn equivalent 3 times less than the reference. Composition like Test4 (Table 2) but added 1 phr of stearic acid. Reduced StA/Zn ratio like in Ref3.	1	1.29

**Table 4:** Heat of melting and peak temperatures of synthesized products and raw materials determined by DSC.

Sample	Phase Change 1		Phase Change 2	
	Heat of Melting [J/g]	Peak [°C]	Heat of Melting [J/g]	Peak [°C]
StZn_s_1	−25.0	57.8	−102.6	121
StZn_s_2	-	-	−95.9	126
StZnS_s_1	−12.7	57.8	−134.2	123
StZnS_s_2	-	-	−108.5	122
StZn_a_1	−11.5	56.1	−162.4	124
StZn_a_2	-	-	−121.9	127
StZnS_a_1_7	−6.3	57.7	−142.3	125
StZnS_a_2_8	-	-	−104.9	123
StZnS_a_1_9	−8.9	56.6	−149.6	123
StZnS_a_2_10	-	-	−104.9	123
StA	−194.5	61.5	-	-
S	−9.1	108.0	−33.5	121
ZnSt	-	-	−153.3	126

**Table 5:** Expected heat of melting calculated from DSC results for selected synthesis products described in Table 1.

Sample	Mass of DSC Sample [mg]	Mass of StA in DSC Sample [mg]	Initial Mass of StA in Sample [mg]	StA Reacted [mg]	ZnSt Yielded [mg]	Expected Heat of Melting [J/g]	Reacted StA [%]
StZn_s_1	11.8	1.52	10.3	8.8	9.8	−127.6	85
StZn_s_2	16.5	0.00	12.8	12.8	14.3	−132.9	100
StZn_a_1	13.6	0.80	11.9	11.1	12.3	−139.4	93
StZn_a_2	14.3	0.00	11.1	11.1	12.4	−132.9	100

**Table 6:** MDR and Mooney test results for compounds described in Table 2 and Table 3 (MDR results: ML—minimum torque; MH—maximum torque; t_s2_—time to 2% rise in torque; MH–ML—torque increment; t_90_—time to 90% rise in torque; Mooney results: ML—minimum viscosity; t_5_—time to increase the viscosity by 5 Mooney units (MU) from the ML value).

Compound	MDR Results					Mooney Results	
	ML [dNm]	MH [dNm]	MH–ML [dNm]	t_s2_ [min]	t_90_ [min]	ML [MU]	t_5_ [min]
Ref0	1.54	7.79	6.25	0.97	1.79	32.8	14.0
Ref1	0.96	9.05	8.09	0.93	1.94	26.1	12.4
Ref2	1.05	9.53	8.48	0.93	2.01	28.3	11.8
Test1	1.17	8.15	6.98	1.00	1.78	31.8	12.7
Test2	1.05	9.21	8.16	1.21	2.88	26.1	18.6
Test3	1.23	8.82	7.59	0.95	1.78	32.0	12.0
Test4	1.10	8.95	7.85	1.10	2.26	28.4	17.2
Test5	1.06	8.02	6.96	0.98	1.74	27.9	12.1
Test6	0.93	8.11	7.18	1.13	2.52	23.4	17.9
Test7	1.18	8.43	7.25	0.96	1.70	29.0	13.0
Test8	1.06	7.47	6.41	1.10	1.88	26.4	16.7
Test9	1.05	8.07	7.02	0.93	1.93	27.8	12.6
Test10	0.99	7.99	7.00	0.88	1.62	27.0	11.4
Test11	1.03	8.32	7.29	0.95	2.27	25.0	16.3
Test12	1.11	8.43	7.32	0.90	1.73	27.9	13.4
Test13	1.04	8.65	7.61	0.86	1.88	27.6	12.8
Test14	1.08	8.11	7.03	0.95	1.80	28.3	12.5
Test15	1.10	8.45	7.35	0.91	1.66	28.8	12.3
Ref3	0.82	6.90	6.08	1.22	3.46	20.9	25.1
Test16	0.98	7.60	6.62	1.32	2.66	22.9	24.1
Test17	1.01	8.24	7.23	1.34	2.87	23.7	24.1
Test18	0.83	6.71	5.88	0.89	2.20	21.9	14.7
Test19	0.94	6.59	5.65	0.75	1.90	24.8	11.6

**Table 7:** Equilibrium swelling and compression set test results for rubber compounds described in Table 2 and Table 3.

Compound	Compression Set [%]			Equilibrium Swelling [%]		
Ref0	72.5	+/−	1.7	96.1	+/−	2.0
Ref1	60.9	+/−	2.3	85.6	+/−	0.4
Ref2	57.9	+/−	1.4	82.6	+/−	1.1
Test1	73.7	+/−	1.1	103.9	+/−	1.9
Test2	52.9	+/−	2.1	74.7	+/−	0.4
Test3	66.8	+/−	1.7	95.2	+/−	0.5
Test4	56.5	+/−	0.9	81.6	+/−	0.5
Test5	74.9	+/−	1.6	105.9	+/−	1.4
Test6	56.3	+/−	1.2	81.3	+/−	1.1
Test7	69.3	+/−	1.1	100.2	+/−	2.0
Test8	61.9	+/−	1.5	93.9	+/−	1.2
Test9	70.4	+/−	0.8	102.1	+/−	2.5
Test10	71.4	+/−	1.6	103.7	+/−	1.4
Test11	68.4	+/−	0.8	92.0	+/−	1.1
Test12	69.2	+/−	1.5	98.9	+/−	1.3
Test13	69.2	+/−	0.2	97.8	+/−	1.3
Test14	70.3	+/−	1.7	102.3	+/−	1.4
Test15	67.9	+/−	1.6	97.1	+/−	0.5
Ref3	44.5	+/−	0.7	82.6	+/−	1.7
Test16	42.5	+/−	2.3	78.3	+/−	1.6
Test17	41.5	+/−	0.8	77.3	+/−	1.4
Test18	51.1	+/−	1.5	85.1	+/−	1.2
Test19	52.7	+/−	0.8	97.1	+/−	1.6

**Table 8:** Tensile test results of the vulcanizates described in Table 2 and Table 3 (SE_100_—stress at relative elongation of 100%, TS—tensile strength, E_b_—elongation at break).

Compound	SE_100_ [MPa]			TS [MPa]			E_b_ [%]		
Ref0	2.6	+/−	0.2	3.0	+/−	0.6	123	+/−	17
Ref1	2.1	+/−	0.1	6.3	+/−	0.3	499	+/−	40
Test2	2.2	+/−	0.1	7.9	+/−	0.7	421	+/−	55
Test4	2.2	+/−	0.1	8.7	+/−	0.1	412	+/−	10
Test6	2.6	+/−	0.1	9.0	+/−	0.1	415	+/−	12
Test8	3.5	+/−	0.2	10.6	+/−	0.4	335	+/−	15
Test9	2.4	+/−	0.2	7.6	+/−	0.1	445	+/−	69
Test14	2.3	+/−	0.1	7.7	+/−	0.1	506	+/−	25
Ref3	2.3	+/−	0.1	8.5	+/−	0.2	435	+/−	8
Test16	2.7	+/−	0.1	8.5	+/−	0.2	352	+/−	23

## Data Availability

The data presented in this study are available on request from the corresponding author due to the confidentiality policy of Hutchinson.

## Supplementary Materials

The following supporting information can be downloaded at https://www.mdpi.com/article/10.3390/polym17212875/s1. Figure S1: Research station used for the synthesis of StA with ZnO and/or S reaction products.

## Abbreviations

The following abbreviations are used in this manuscript:

ZnSt	Zinc stearate
EPDM	Ethylene–propylene–diene monomer
ZnO	Zinc oxide
StA	Stearic acid
LCA	Life Cycle Analysis
GHS	Globally Harmonized System of Classification and Labelling of Chemicals
CLD	Caprolactam disulfide
MBTS	2,2’-benzothiazyl disulfide
CBS	N-cyclohexyl-2-benzothiazole sulfenamide
ZDTP	Zinc dialkyldithiophosphate
MDR	Moving Dye Rheometer
CS	Compression set
FTIR	Fourier Transform Infrared Spectroscopy
DSC	Differential Scanning Calorimetry
SEM	Scanning Electron Microscopy
TD-GC-MS	Thermal Desorption–Gas Chromatography–Mass Spectrometry
EDS	Energy-Dispersive X-ray Spectroscopy
