# Supplementary figures and images for: The Influence of Zinc Stearate Complexes on the Sulfur Vulcanization of Ethylene–Propylene–Diene Monomer
Source: Polymers (Basel). 2025 Oct 28;17(21):2875. doi: 10.3390/polym17212875 (PMC12611030; doi:10.3390/polym17212875)

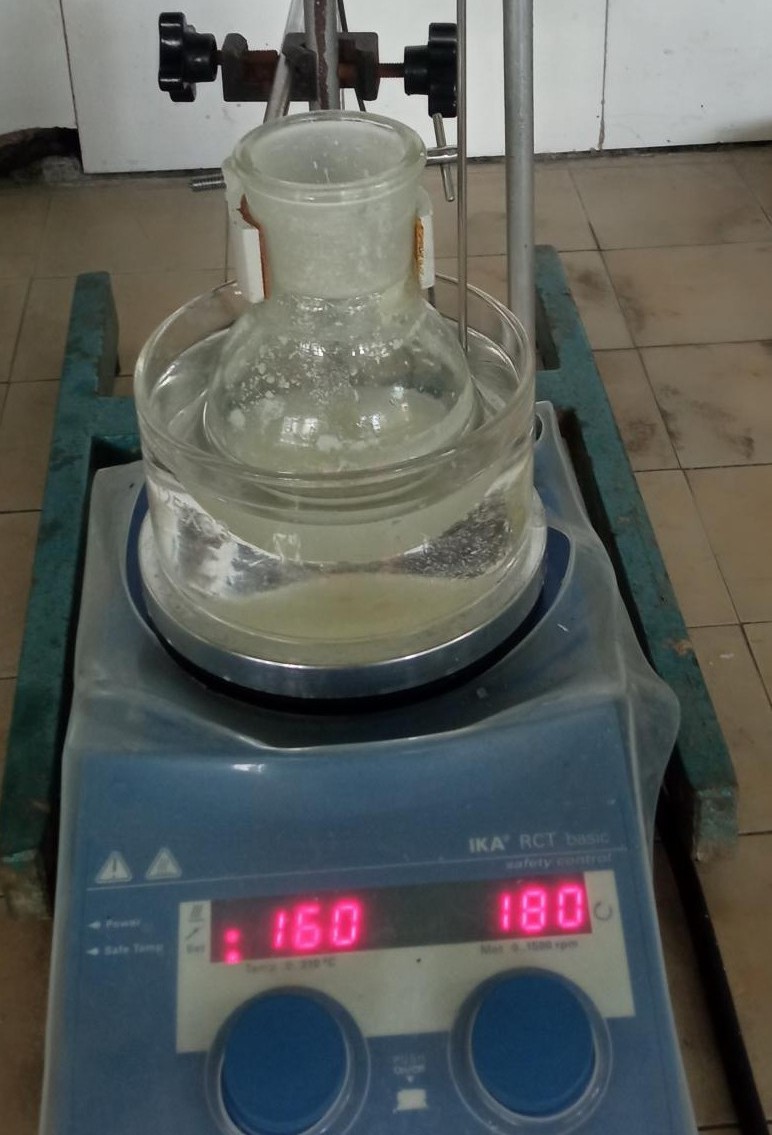

Supplement: Supplementary file 1 [file polymers-17-02875-s001.zip › polymers-3896756-supplementary.jpg]
